# The effect of six days of dietary nitrate supplementation on performance in trained CrossFit athletes

**DOI:** 10.1186/s12970-016-0150-y

**Published:** 2016-11-03

**Authors:** Samuel J. Kramer, Daniel A. Baur, Maria T. Spicer, Matthew D. Vukovich, Michael J. Ormsbee

**Affiliations:** 1 Department of Nutrition, Food and Exercise Sciences, Institute of Sports Sciences and Medicine, Florida State University, 1104 Spirit Way, Tallahassee, FL 32306 USA; 2 Department of Health & Nutritional Sciences, South Dakota State University, Brookings, SD USA; 3 Discipline of Biokinetics, Exercise, and Leisure Sciences, University of KwaZulu-Natal, Durban, South Africa

**Keywords:** Potassium nitrate, Rowing, Supplementation, Beetroot, Anaerobic power

## Abstract

**Background:**

While it is well established that dietary nitrate reduces the metabolic cost of exercise, recent evidence suggests this effect is maintained 24 h following the final nitrate dose when plasma nitrite levels have returned to baseline. In addition, acute dietary nitrate was recently reported to enhance peak power production. Our purpose was to examine whether chronic dietary nitrate supplementation enhanced peak power 24 h following the final dose and if this impacted performance in a heavily power-dependent sport.

**Methods:**

In a double-blind, randomized, crossover design, maximal aerobic capacity, body composition, strength, maximal power (30 s Wingate), endurance (2 km rowing time trial), and CrossFit performance (Grace protocol) were assessed before and after six days of supplementation with nitrate (NO) (8 mmol·potassium nitrate·d^−1^) or a non-caloric placebo (PL). A 10-day washout period divided treatment conditions. Paired *t*-tests were utilized to assess changes over time and to compare changes between treatments.

**Results:**

Peak Wingate power increased significantly over time with NO (889.17 ± 179.69 W to 948.08 ± 186.80 W; *p* = 0.01) but not PL (898.08 ± 183.24 W to 905.00 ± 157.23 W; *p* = 0.75). However, CrossFit performance was unchanged, and there were no changes in any other performance parameters.

**Conclusion:**

Consuming dietary nitrate in the potassium nitrate salt form improved peak power during a Wingate test, but did not improve elements of strength or endurance in male CrossFit athletes.

## Background

Nitric oxide is a signaling molecule which contributes to numerous physiological functions including mitochondrial respiration and biogenesis, vasodilation, muscle glucose uptake, angiogenesis, and sarcoplasmic reticulum calcium handling [1]. As these processes support various physical functions, maximizing nitric oxide production and availability may support exercise performance. Importantly, nitric oxide production can be augmented by ingestion of foods containing nitrate, a precursor for nitrite, which is ultimately reduced to nitric oxide [2].

Elevations in plasma nitrite levels via dietary nitrate have been associated with enhanced endurance capacity and performance [3–8]. These results likely occur via dietary nitrate-mediated reductions in the metabolic cost of exercise. Interestingly, it was recently reported that reductions in the metabolic cost of exercise were evident with chronic nitrate supplementation (6 mmol·d^−1^; 7–30 day) 24 h following the final nitrate dose even though plasma nitrite values had returned to baseline levels [9]. As this may help to guide practical supplementation strategies for athletes, more research is warranted to determine whether this residual effect translates to performance benefits.

In addition, recent research suggests that acute dietary nitrate ingestion augments power production. Coggan et al. [10] reported enhanced isokinetic knee extensor power and calculated velocity 2 h following ingestion of 140 mL of beetroot juice (~11.2 mmol of nitrate) in healthy men and women. Similarly, Rimer et al. [11] recently reported that acute (2–3 h preceding exercise) beetroot juice ingestion (140 mL; ~11.2 mmol nitrate) enhanced peak cycling power and pedaling velocity in endurance and power-trained athletes. However, whether chronic nitrate supplementation enhances peak power production is yet to be determined. Moreover, no investigations have assessed whether there is a residual effect of chronic nitrate supplementation on peak power production ≥24 h following the final nitrate dose.

Finally, the impact of dietary nitrate on sport-specific performance among power athletes is yet to be fully determined. CrossFit is a sport that includes a wide variety of generally short-duration, high-intensity exercises ranging from Olympic weight lifting to rowing exercises. Performance in this sport is highly dependent on peak power and fatigue resistance [12]. As such, dietary nitrate may be beneficial for CrossFit athletes.

Therefore, our purpose was to investigate the impact of chronic (6 days) nitrate supplementation on laboratory-based and sport-specific performance outcomes among CrossFit athletes ≥24 h following the final nitrate dose. We hypothesized that nitrate supplementation would enhance peak power, 2K rowing performance, and CrossFit performance in these athletes.

## Methods

### Participants

Male CrossFit athletes (*N* = 12) aged 20–35 years were recruited to participate in this study. Characteristics are outlined in Table 1. Participants were not taking any other supplements at the time of the study with the exception fish oil, protein and/or multivitamins. The participants were required to be training in a CrossFit facility ≥ 3 days·week^−1^ and for ≥ 4 months. Subjects were encouraged to maintain normal training for the duration of the study (~40 days).

**Table 1:** Participant characteristics (*N* = 12)

Variables	Values
Age (yr)	23 ± 5
Height (cm)	175.9 ± 7.4
Body Mass (kg)	82.7 ± 13.5
Fat Free Mass (kg)	70.6 ± 7.2
Fat Free Mass (%)	86.1 ± 5.7
Fat Mass (kg)	12.1 ± 7.8
Fat Mass (%)	13.9 ± 5.7
VO_2Peak_ (ml·kg^-1^·min^−1^)	48.5 ± 7.0

Values are present mean ± standard deviation

*VO*_*2Peak*_, peak oxygen consumption

### Research design

**Fig. 1:**
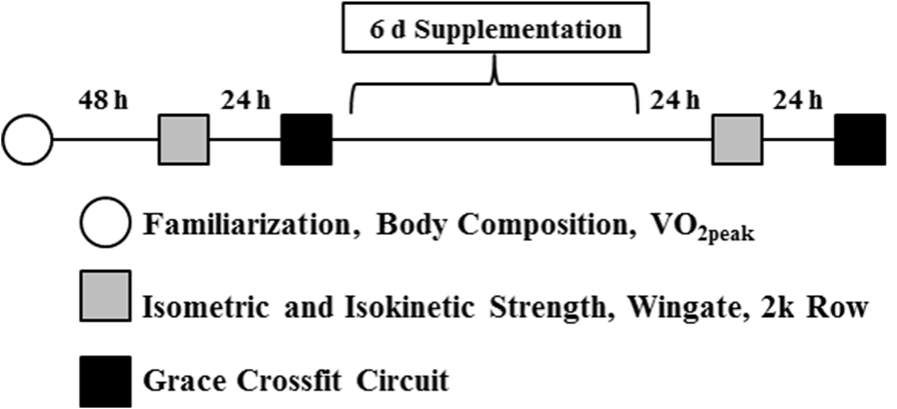
Timeline of events of the experimental protocol for either nitrate or placebo group. The protocol lasted for approximately 40 days total. Note: After washout period, the protocol was repeated using the opposite supplement originally assigned

The study design is outlined in Fig. 1. In this randomized, double-blind, crossover study, two treatments were ingested for 6 days: 1) 8 mmol·d^−1^ potassium nitrate (NO; 2 × 4 mmol capsules, one consumed in the morning, one in the evening) or 2) 8 mmol·d^−1^ nitrate-free potassium chloride (PL) (2 × 4 mmol capsules, one consumed in the morning, one in the evening). Both NO and PL were prepared by the Shaklee Corporation®. The final nitrate dose was consumed ≥24 h prior to performance testing (see Fig. 1).

Time of day for all testing was kept consistent (±2 h) for each subject to prevent circadian variation. Participants arrived fasted for at least 3 h on all testing days. Water was allowed *ad libitum*. During the tests, no music, encouragement or motivation was provided and participants were prohibited from seeing their results and/or time to complete certain testing procedures, but they were permitted to see how much distance, repetitions, or time remained for respective tests.

During the study period, participants were instructed to follow their normal dietary habits, but were provided with a list of foods high in dietary nitrate and asked to abstain from these foods throughout the duration of the study. Prior to the study, participants filled out a 72 h food log (2 weekdays, 1 weekend day). A second 72 h food log was filled out at the end of the entire study to track any dietary changes. A copy of pre-testing food logs was given back to participants to facilitate the replication of dietary intake patterns each day preceding testing. The participants were asked to avoid strenuous exercise, alcohol, and caffeine 24 h prior to each testing session. Furthermore, participants were requested to abstain from using antibacterial mouthwash and chewing gum as these are known to negatively impact production of nitric oxide [13]. Participants were also asked to arrive at each testing session adequately hydrated (i.e. without subjective feelings of thirst). Participants were allowed to use exercise aids (excluding chalk) such as tape or wrist wraps for the CrossFit specific “Grace” test, but had to utilize them for all of the trials throughout the study for consistency.

### Anthropometrics, body composition, maximal aerobic capacity and familiarization

Participants visited the laboratory 48 h prior to baseline performance testing. During this session, height (to the nearest 0.1 cm) and weight (to the nearest 0.1 kg) were obtained with the use of a wall-mounted SECA 216 stadiometer (SECA®, Hamburg, Germany) and a digital scale (Detecto® 750, Webb City, MO). Then, body composition was determined using air displacement plethysmography with thoracic volume being estimated (BodPod®, Cosmed USA Inc., Chicago, IL). Percent body fat was estimated via the Siri equation [14]. Thoracic volume was estimated based on reference data in healthy adults [15]. These measurements were taken without shoes, and wearing only compression shorts. Finally, a peak oxygen consumption (VO2_peak_) test on a motor driven treadmill (Woodway®, Waukesha, WI, USA) was performed with a calibrated metabolic cart (TrueOne 2400, Parvo Medics, Inc., Sandy, UT, USA). The speed of the treadmill was set at 9.66 km·h^−1^ at 0 % grade. The grade was then increased by 2 %·min^−1^ until volitional exhaustion. The sign for volitional exhaustion was when the participants could no longer keep pace with the treadmill belt, and/or they indicated to the researcher with a “thumbs down” hand signal that they desired to stop the test. After this preliminary testing, participants were familiarized with all procedures on one occasion by completing all testing protocols without data being collected.

### Visit 1

After arrival at the laboratory, isokinetic and isometric extension and flexion strength testing was performed on a dynamometer (Biodex System 3, Biodex Medical Systems, Inc., Shirley, New York). The participants were seated with their dominant leg strapped to the lever arm at a 90°. To further secure participants, a lap belt and two crossing shoulder belts over the chest were affixed. The participants were asked to cross their arms and keep their hands flat against the chest belts so as not to induce isopressor increases in blood pressure or alter leverage to enhance force production. On the dynamometer, the isokinetic quadriceps extension and hamstring flexion contractions consisted of 2 × 5 repetitions at both 60°·s^−1^ and 180°·s^−1^ with 1 min rest between each set. The isometric contractions consisted of 2 × 5 quadriceps extension and hamstring flexion repetitions and were performed at 60° of knee flexion with 5 s rest between repetitions and 1 min rest between sets. Subjects were not provided verbal encouragement or performance feedback.

After 10 min of passive rest, maximal power and fatigability was measured via a 30 s Wingate test on a Velotron cycle ergometer (RacerMate, Inc., Seattle, WA). Testing procedures have been described elsewhere [16]. The participants were asked to remain seated during the test.

After a 15-min passive rest in which the participants could drink water *ad libitum*, participants mounted a rowing ergometer (Concept2® Model D, Concept2, Inc., Morrisville, VT), and their feet were strapped down via Velcro straps. Participants then completed a 3 min warm-up consisting of 10 subjectively “hard” strokes at the start of each min. Thereafter, a 2K time trial was completed as quickly as possible at a resistance level of 3–5. During the warm-up and time trial, participants were monitored to ensure adequate form. A good stroke consisted of the body progressing forward up the slide as far as possible and then driving back with the legs, back and arms, in that order, at the top of each stroke. Relative time was calculated from a weight-adjustment factor provided by the ergometer manufacturer:

$$ {\left[ body\  mass\  in\  pounds/ 270\right]}^{0.222}x\  raw\  time\ (s) $$

### Visit 2

The next day, the CrossFit workout known as “Grace” was performed. The Grace is a common benchmark workout in CrossFit used to track performance improvements. This performance test was chosen due to its simplicity relative to other CrossFit benchmarks (i.e. one movement and one apparatus), and because of its heavy dependence on power development and endurance. As it is commonly featured in CrossFit competitions, the Grace was taken to be a valid assessment of CrossFit performance. For the Grace performance test, each participant was instructed to complete 30 clean-and-jerk repetitions using 61.37 kg as fast as possible. The total time to complete all 30 repetitions was recorded and used for data analysis on each trial. The participants were allowed to warm up with the bar by itself and with weights if desired, with a limit of 61.37 kg. A complete repetition included the elbows being locked out when the bar came above the head and plates touching the ground when bringing the bar downwards for the next repetition.

### Supplementation protocol

Participants consumed their assigned supplement for 6 days (beginning the evening following Visit 2 testing). The participants took one pill in the evening, 30–60 min before bed each night and another pill 30–60 min before they ate their first meal of the day each morning. The supplement protocol was similar to previous research [17, 18] that tested the effects of dietary nitrate supplementation for 15 days and reported increases in blood nitrate following supplementation. Empty supplement bottles were returned to the research staff to verify compliance after 6 days of supplementation. During the supplementation period, the participants were only allowed to exercise for the first 4 days, but could not exercise at all on days 5 and 6. Compliance was confirmed with a training log and by a weekly mid-point phone call to remind participants of the supplementation and training instructions. After the 6 days supplementation period, participants returned to the laboratory for post-testing (Visit 3 and 4). Following a 10 day washout period (common among similar studies [5, 6]), participants were assigned the opposite treatment that they took for the first 6 days, and repeated the same procedures described above.

### Dietary analysis

All pre-test and post-test dietary food logs were analyzed by the same researcher using The Food Processor software (ESHA Research®, V.10.11, Salem, OR).

### Statistical analysis

Treatment order was randomized using an internet-based application (Random Sequence Generator, www.random.org). Changes from pre- to post-treatment were examined via a paired *t*-test. Additionally, change scores (∆) were calculated and compared via paired student’s *t*-test. All analyses were completed on SPSS version 22 (IBM Corp., Armonk, NY). Values were presented as mean ± SD.

## Results

### Strength, power, and endurance testing

**Fig. 2:**
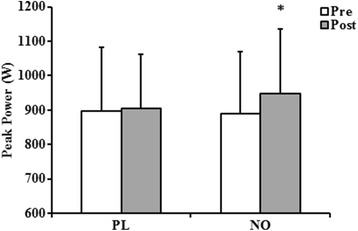
Changes in peak power during a Wingate test pre to post 6 day of placebo or nitrate supplementation. PL, placebo; NO, nitrate. Data are presented as mean ± standard deviation. * denotes an increase in peak power relative to pre-treatment (*p* = 0.01)

Strength, power, and endurance testing results are presented in Table 2. There were no differences from pre- to post-treatment nor between ∆ for any strength, power, or endurance variable with one exception. For the 30-s Wingate, there was a significant increase in peak power following supplementation with NO (*p* = 0.01; Fig. 2). Moreover, there was a strong trend for a larger ∆ with NO relative to PL (*p* = 0.08).

**Table 2:** Effects of nitrate supplementation on performance among CrossFit athletes

Performance Test	PL			NO		
	Pre	Post	Δ (%)	Pre	Post	Δ (%)
Grace Test (s)	281.75 ± 41.84	270.92 ± 129.16	−3.84 ± 8.94	295.92 ± 170.98	263.67 ± 117.74	−8.94 ± 31.14
Isometric Ext.60° (*N*)	174.15 ± 27.59	184.80 ± 43.41	6.11 ± 17.36	169.06 ± 36.63	186.29 ± 48.67	10.19 ± 36.60
Isometric Flex. 60° (*N*)	119.83 ± 16.62	125.53 ± 19.66	4.76 ± 18.29	116.82 ± 21.35	118.95 ± 26.51	1.82 ± 24.17
Isokinetic Ext. 60° · s^−1^ (*N*)	184.07 ± 48.53	179.31 ± 44.09	−2.59 ± 9.15	174.61 ± 41.03	167.76 ± 50.14	−3.92 ± 22.20
Isokinetic Flex. 60° · s^−1^ (*N*)	106.40 ± 24.54	103.59 ± 25.12	−2.64 ± 2.36	103.73 ± 21.32	102.19 ± 26.41	−1.48 ± 23.87
Isokinetic Ext. 180° · s^−1^ (*N*)	120.14 ± 42.32	123.28 ± 35.31	2.68 ± 16.56	120.33 ± 35.98	128.21 ± 31.95	6.55 ± 11.20
Isokinetic Flex. 180° · s^−1^ (*N*)	75.65 ± 27.59	76.23 ± 20.43	0.77 ± 25.95	79.35 ± 14.17	79.68 ± 16.00	0.42 ± 12.91
Wingate Peak (W)	898.08 ± 183.24	905.00 ± 157.23	0.77 ± 14.19	889.17 ± 179.69	948.08 ± 186.80^a^	6.62 ± 3.96
Wingate Mean (W)	703.08 ± 101.66	736.08 ± 95.31	4.69 ± 6.25	724.17 ± 109.93	737.58 ± 118.93	1.85 ± 8.19
2 K TT (s)	465.07 ± 28.45	459.87 ± 24.85	−1.12 ± 12.65	457.57 ± 23.56	459.73 ± 23.93	0.47 ± 1.57
2 K TT (s · kg^−1^)	392.43 ± 79.51	387.26 ± 71.09	−1.31 ± 10.59	384.78 ± 66.59	387.40 ± 72.44	0.68 ± 8.78

Values are present mean ± standard deviation; *PL* placebo, *NO* nitrate, *s* seconds, *W* watts, *N* newtons, *TT* time trial, *Ext.* extension; *Flex.* Flexion. Grace is defined as 30 clean-and-jerks, and performance is defined as time to complete all repetitions. All isometric and isokinetic tests evaluate peak torque

^a^indicates increase from pre- to post-supplementation as determined via paired *t*-test, *p* = 0.01

### CrossFit performance

Results are outlined in Table 2. There were no changes in CrossFit performance.

### Adverse effects

Adverse events were reported for both NO (*n* = 5) and PL (*n* = 3). Slight nausea and gastrointestinal discomfort were reported with morning supplement consumption in NO, but these subsided once food was consumed. Similarly, slight gastrointestinal discomfort was reported with morning supplement consumption in PL, but these subsided once food was consumed.

## Discussion

Previous research has established that dietary nitrate reduces the metabolic cost of exercise, and this effect is maintained 24 h following the final dose of a nitrate supplementation regimen [9]. Additionally, nitrate supplementation has been reported to enhance peak power output [10, 11]. However, whether this ergogenic effect is also present following 24 h following nitrate ingestion, or if this ergogenic effect translates to performance improvements in sport-specific tasks dependent on power has not been determined. The primary finding of the current study was that 6 days of potassium nitrate supplementation enhanced peak power output in a Wingate cycling test among trained CrossFit athletes when assessed 24 h following the final nitrate dose. However, there were no improvements in any other performance outcome including time to complete a Grace CrossFit workout.

In the current study, nitrate supplementation enhanced peak power production. This is the first study to report enhanced peak power as a result of chronic dietary nitrate supplementation 24 h following the final nitrate dose. Prior studies have reported enhanced peak power following acute nitrate supplementation [10, 11]. Specifically, Coggan et al. [10] reported enhanced isokinetic knee extensor power among healthy men and women 2 h following 140 mL (~11.2 mmol nitrate) of beetroot juice. Additionally, Rimer et al. [11] reported enhanced peak power in a Wingate test among endurance and power-trained athletes following the same 140 mL beetroot juice dose. There are a number of possible mechanisms for enhanced Wingate peak power with chronic nitrate supplementation (discussed below).

A novel finding of the current study was that the effects of chronic nitrate supplementation on peak power were maintained ≥24 h following the final nitrate dose. Wylie et al. [9] recently found that ~28 days nitrate supplementation (6 mmol·d^−1^) reduced the metabolic cost of exercise 24 h following the final nitrate dose despite plasma nitrite levels returning to baseline. Our data suggests that chronic nitrate-mediated effects on peak power are similarly maintained 24 h following the final nitrate dose. Prior research has indicated that nitrate supplementation causes increased production of mouse skeletal muscle structural proteins involved in calcium handling [19]. These changes may be maintained for a period of time following the reduction in plasma nitrite post-supplementation. Additionally, skeletal muscle and/or other tissues may act as a reservoir for nitric oxide precursors like nitrate and nitrite, and storage of these products may be maintained ≥ 24 h [20]. Interestingly, these “residual” changes in peak Wingate power 24 h following the final nitrate dose were apparent following only 6 days of nitrate supplementation. In contrast, Wylie et al. [9] did not report changes in the metabolic cost of exercise following 7 days of supplementation, but did see a reduction following ~28 days. It is possible that structural changes which influence peak power are unique from those that impact the metabolic cost of exercise and are more sensitive to nitrate supplementation. However, more research is warranted to elucidate these mechanisms.

There were no changes in peak torque during isokinetic testing. This finding is in contrast to a prior study [10]. Coggan et al. [10] assessed isokinetic knee extensor torque at 0°·s^−1^, 90°·s^−1^, 180°·s^−1^, 270°·s^−1^, and 360°·s^−1^. It is possible that we failed to detect any changes in isokinetic peak torque due to the velocities utilized in the current study (60° · s^−1^ and 180° · s^−1^). In the study by Coggan et al. [10], power was significantly enhanced at 360°·s^−1^, but at no other velocities. This data suggests that the impact of nitrate on peak torque may only apparent at high velocities, which may explain why we failed to note any differences for isokinetic knee extensor torque in the current study. Prior research in animals suggests that nitrate supplementation may augment maximal shortening velocity in fast-twitch muscle fibers, but have relatively little impact on slow-twitch fibers [21, 22]. If this is also the case in humans, it makes sense that peak power in a Wingate test, but not isokinetic power at 60°·s^−1^ and 180°·s^−1^, would be enhanced following nitrate supplementation as this exercise is likely highly reliant on fast-twitch fibers [23].

Despite enhancements in peak power output in the Wingate test, performance in a 2 km rowing time trial was unchanged. This contrasts with recent research investigating the effects of nitrate on high-intensity exercise capacity. For instance, a number of studies have reported enhanced performance in short-duration (~6 min), high-intensity time trials [4, 24], endurance capacity in exercise lasting ~5–12 min [5, 8] and performance in high-intensity intermittent running [25]. Worth noting, two recent studies have reported enhanced performance among well-trained rowers following acute or chronic nitrate supplementation [24, 26]. Hoon et al. [24] found 2K rowing performance to be possibly enhanced with 140 mL of beetroot juice (8.4 mmol nitrate) 2 h prior to exercise. Moreover, Bond et al. [26] reported enhancements in time to complete 6 repeated 500 m rowing bouts following 6 days of beetroot juice supplementation (5.5 mmol nitrate·d^−1^) in well-trained junior rowers. Based on these studies, our finding of no change in time trial performance is somewhat surprising, particularly considering that our subjects were familiarized, and the exercise protocol seemed to be highly reliable (<1.5 % CV) even for athletes who were less-accustomed to rowing exercise. It is possible that the 24 h delay between the final nitrate dose and the time trial influenced our findings despite the aforementioned prior study [9] reporting that nitrate-mediated improvements in exercise efficiency are maintained within this timeframe. Another possibility is that the form of nitrates consumed influenced performance effects. Indeed, a recent study [27] showed that the oxygen cost of exercise was reduced with pre-exercise beetroot juice, but not nitrate salt, ingestion.

Performance was also unchanged in the Grace CrossFit protocol. One other study has investigated CrossFit specific workouts following supplementation (6 weeks) with a product that contains nitrate (beetroot extract) [16]. In this study, two CrossFit workouts were assessed for changes from pre- to post-supplementation: 1) a 500-m row, 40 wall balls, 30 push-ups, 20 box jumps, and 10 thrusters, and 2) 800 m run followed by as many rounds as possible of 5 burpees, 10 Kettlebell swings, and 15 air squats within 15 min. Similar to our results, supplementation did not provide a statistically significant difference in terms of improvement for the first CrossFit workout. However, in agreement with this prior study, we found large magnitude relative changes in time to complete the workouts with NO vs. PL (−8.94 % ± 31.14 % vs. −3.84 ± 8.94 %), which were suggestive of benefits. Of note, the prior study [16] did report a likely increase in repetitions completed in the second workout suggesting that supplementation with products containing nitrate benefit CrossFit performance; however, this is somewhat speculative as beetroot extract was only one of a number of other ingredients contained in the supplement utilized. Taken together, our results combined with this prior study indicate potential benefits of nitrate on CrossFit performance, but future research is warranted to determine more reliable methods of assessing performance changes in this sport.

A number of instances of gastrointestinal distress were reported with nitrate supplementation in the current study. This may have been a consequence of the nitrate form utilized (i.e. nitrate salt). Indeed, gastrointestinal symptoms have been reported in a prior study utilizing nitrate salts [28]. As such, it may be advisable for athletes to consume nitrate in whole food forms such as beetroot juice particularly in light of the recent evidence suggesting this has a greater impact on the metabolic cost of exercise versus nitrate salts [27].

This study had several limitations. First, CrossFit performance testing was conducted ~48 h following the final dose of supplement in an effort to avoid the influence of gastrointestinal distress on performance. As such, this may have attenuated any nitrate-mediated performance benefits. In addition, performance results with the CrossFit protocol utilized in the current study were highly variable. The Grace protocol requires all participants to lift the same amount of weight (61.4 kg) despite sometimes drastic differences in body mass and strength. As a result, time to complete the Grace protocol varied considerably between participants. Moreover, in participants who were particularly challenged by the weight, within-subject variation was also high due to failed lifts. Thus, while the Grace is a common benchmark workout in CrossFit, it may lack sensitivity for small, but meaningful changes. Lastly, participants in the study only completed one familiarization session of the all the exercise protocols. As such, it is possible that learning effects were present, particularly with isokinetic strength testing [29]. However, studies specifically examining the reproducibility of Biodex isokinetic strength over multiple sessions have found no evidence of a learning effect [30, 31]. Thus, we are confident that our familiarization was sufficient to prevent learning effects.

## Conclusion

In conclusion, 6 days of dietary nitrate supplementation in male CrossFit athletes improved peak Wingate power 24 h following the final nitrate dose. However, there were no improvements in rowing time- trial or CrossFit performance. Future research is warranted to elucidate mechanisms responsible for changes in peak power output, improve methods for assessing CrossFit performance, and further investigate whether nitrate supplementation enhances performance in strength and power-based sports.

## Abbreviations

2K: 2000-meter rowing test
ADP: Adenosine diphosphate
BRJ: Beetroot juice
eNOS: Endogenous nitric oxide synthase
N: Newtons
NO: Potassium nitrate
PCr: Phosphocreatine
PL: Placebo
